# Autonomous motivation, social support, and physical activity in school children: moderating effects of school-based rope skipping sports participation

**DOI:** 10.3389/fpubh.2024.1295924

**Published:** 2024-01-24

**Authors:** Yufei Qi, Yao Yin, Xuyang Wang, Yong Zou, Bo Liu

**Affiliations:** ^1^Department of Physical Education and Research, Central South University, Changsha, China; ^2^Progression School of Upper Secondary, Beijing College of Finance and Commerce, Beijing, China; ^3^Xiangya School of Medicine, Central South University, Changsha, China; ^4^Logistics Department, Central South University, Changsha, China; ^5^Institute of Basic Education, Hefei Technology College, Hefei, China; ^6^Faculty of Physical Education, Srinakharinwirot University, Bangkok, Thailand

**Keywords:** interaction, moderate to high intensity physical activity, structural equation modelling school children, rope skipping, school children

## Abstract

**Purpose:**

Recent studies have shown that physical activity (PA) levels are low among children and adolescents globally. In order to reverse this trend, PA interventions are increasingly favoured. The school setting is the ideal place to address the issues that many children face. The purpose of this study was to (a) The primary focus of this study is to delve into the mediating role played by school-based rope skipping sports participation (SRSP) in the connection between social support and moderate to high-intensity physical activity (MVPA) among school children. (b) Additionally, this research aims to examine the moderating effect of within this pathway.

**Methods:**

We conducted a survey involving 721 adolescents residing in Changsha City. The participants’ ages ranged from 8 to 12 years, with an average age of 9.84 ± 1.535 years. Out of these participants, 406 were boys, and 315 were girls. To assess variables such as social support and autonomous motivation, we employed standardized measurement scales. Subsequently, we analyzed the collected data using various statistical methods, including independent s-amples t-tests, bivariate correlation analysis, descriptive statistical analysis, structural equation modeling (SEM), and the Johnson-Neyman method.

**Results:**

An independent samples t-test revealed a statistically significant difference in MVPA between genders (*p* = 0.003 < 0.05), with boys exhibiting a higher level of engagement in MVPA compared to girls, Correlation analysis revealed significant positive associations among several key variables. Specifically, social support demonstrated a noteworthy positive correlation with autonomous motivation (r = 0.331, *p* < 0.01) as well as school children’s engagement in MVPA (r = 0.308, p < 0.01). Moreover, autonomous motivation displayed a significant positive correlation with school children’s involvement in MVPA (r = 0.459, *p* < 0.01). The moderating analysis revealed a significant influence of the interaction between increased participation in and social support on school children’s engagement in MVPA.

**Conclusion:**

Social support and autonomy support have been proven effective in enhancing school children’s engagement in MVPA. They exert their influence indirectly by fostering autonomous motivation. Notably, robust social support can significantly benefit MVPA school children with high activity requirements, particularly those regularly engaged in MVPA during the school day.

## 1 Introduction

In our rapidly evolving modern society, significant shifts in lifestyles have led to a concerning trend among school children—an insufficient level of physical activity (PA), especially in the realm of moderate to high-intensity physical activity (MVPA) (1, 2). This trend gives rise to a host of issues affecting their physical and mental well-being (3, 4). Research has compellingly demonstrated the intricate connection between school children’s engagement in MVPA and their overall health, social aptitude, academic performance, and mental health (5–7). Participation in MVPA offers a multitude of advantages for school children, including enhancements in cardiorespiratory fitness, muscle strength, bone development, psychological well-being, and cognitive abilities (8–11). Regrettably, the advent of modern technology and shifts in societal dynamics have profoundly transformed the lifestyles of school children. The widespread prevalence of technological devices like televisions, mobile phones, and computers has lured them into spending more time on indoor electronic leisure pursuits, leaving limited room for outdoor sports and PA (12, 13). Simultaneously, escalating academic pressures and the weighty burden of schoolwork have compelled school children to allocate more of their time to studies, subsequently reducing the time available for physical exercise (14). These converging factors have precipitated a decline in the PA levels of school children, and the adverse repercussions on their health and development are gradually becoming increasingly evident (15).

Youth participation in PA is not only determined by individual characteristics and choices, but more importantly is related to environmental factors. The campus environment is being influenced by some of the options related to exercise and the various opportunities to use physical education time for exercise (16).

It has been suggested that the school environment explains an important part of the variation in PA in school children (17, 18). In addition, the inability of school children to carry out or participate in activities at school is an important factor affecting their participation in PA (19). Therefore, PA facilities and the provision of extra-curricular activities in schools are key factors to be considered in promoting PA among school children.

Schools, being the primary environment for school children, bear the crucial responsibility of nurturing their comprehensive development (20). Within this framework, physical education stands out as a pivotal avenue for providing these students with opportunities for PA (21). Nonetheless, the degree to which school children engage in MVPA is subject to numerous influencing factors, with autonomous motivation and social support emerging as vital moderating elements (22, 23). Research has convincingly underscored the importance of self-regulation and external support in fostering MVPA among school children (24). Autonomous motivation, representing an intrinsic form of motivation, encompasses both autonomous intrinsic motivation and autonomous extrinsic motivation (25). Autonomous intrinsic motivation stems from an individual’s genuine interest in an activity and their sense of personal value, Autonomous intrinsic motivation is the only predictor of moderate-intensity physical activity in school children (26), while autonomous extrinsic motivation is rooted in external rewards and acknowledgment (25). The level of autonomous motivation exhibited by school children directly relates to their motivation, persistence, and effectiveness in engaging in PA (27). Moreover, social support plays an instrumental role in influencing MVPA among school children (28). Social support (SS) is a key reinforcer of the children and young people’s PA promotion (YPAP) model, and participation in PA with a range of supportive behaviors is essential to promote PA in children and young people (29). Social support encompasses emotional understanding, encouragement, recognition, and the provision of practical feedback, assistance, and resources (17). At the same time, social support may influence autonomous motivation in school children. Previous research suggests that autonomy support from a significant other may influence a person’s motivation to be autonomous (30). When school children receive guidance and support from their social circles during PA, it significantly bolsters their motivation and drive to participate. Nonetheless, it is imperative to identify integrated measures that can synergistically harness the powers of autonomous motivation and social support to foster PA among school children (31). Therefore, there is a need to better understand the factors influencing school children’s participation in PA and the mechanisms involved in order for interventions to provide insights.

School-based rope skipping sports participation (SRSP) offers a straightforward, cost-effective, and inclusive approach to PA, with distinct advantages (32). Through rope skipping exercises, school children can enhance their cardiorespiratory function, boost muscle strength and coordination, improve physical fitness, foster teamwork, and cultivate perseverance (33, 34). It boasts a low entry barrier, making it easy for school children of varying ages and physical fitness levels to engage in, and is well-suited for widespread adoption in schools (35). Research has demonstrated that not only bolsters school children’s enthusiasm for sports but also elevates their engagement in MVPA (36). This suggests that SRSP might encourage participation among school children due to its affordability, accessibility for families of lower socio-economic backgrounds, and its interactive nature, which garners support from peers in the same class (35). However, limited research has explored the moderating effects of SRSP. Therefore, this study aims to investigate the moderating influence of SRSP on the relationship between autonomous motivation and social support, as well as MVPA among school children. It seeks to construct a conceptual model using a sample of school children to shed light on these dynamics.

In summary, this study tested a structural equation modelling (SEM) with a sample of school children. The study hypothesised that (1) there is a positive correlation between social support and autonomous motivation and MVPA in school children, effectively increasing MVPA engagement. (2) Social support enhances school children’s autonomous motivation, thereby increasing MVPA. i.e., autonomous motivation mediates the relationship between social support and MVPA. (3) SRSP moderates’ school children’s autonomous motivation and social support and the relationship between the two and MVPA. Our aim was to examine in depth the relationships between autonomous motivation, social support, and MVPA in school children. In addition, we aimed to investigate the moderating effects of these relationships. By analysing these factors in depth, we hope to lay a scientific foundation for strengthening physical education in schools and promoting the overall health and development of school children.

## 2 Research methodology

### 2.1 Study design and participants

Between September and December 2022, we conducted comprehensive testing and surveys. To ensure a representative sample, we employed a whole cluster random sampling method, taking into account both urban and suburban areas. Specifically, we selected six key urban districts in Changsha City, Hunan Province, and identified one primary school (comprising grades 3 to 5) in each district. Within these selected schools, we randomly designated one class per grade as our research subjects. This process resulted in a total of 20 classes, encompassing 800 students. Following the principle of voluntary participation, our research team distributed informed consent forms to both students and their caregivers. These documents outlined the study’s purpose, procedures, potential benefits, and any inconveniences that might arise. Ultimately, 750 students and their caregivers voluntarily signed the informed consent form to participate in the study. To assess PA levels, we employed accelerometers to monitor the PA of these 750 students over a one-week period. Additionally, we administered questionnaires to both the students and their caregivers. Out of the 750 questionnaires distributed, 735 were returned. After careful evaluation, 706 of these were deemed valid, resulting in a robust 96% valid return rate.

### 2.2 Data acquisition

#### 2.2.1 Accelerometer

MVPA time was measured using a GT3X Human Movement Energy Monitor (ActiGraph, Pensacola, FL). Accelerometers are highly effective instruments for assessing PA levels and estimating energy expenditure in children and adolescents (37). Their widespread utilization is evident in numerous national and internation. During the study, school children were instructed to wear the accelerometers securely fastened to their hips continuously for seven consecutive days. The device should be worn for a minimum of 10 h per day, as the definition of the effective wearing time will have an impact on the PA measurements, and the devices were only removed during water-related activities and sleep. Data collection, initialization of the devices, data retrieval, and data processing were all conducted using ActiLife software (version 15.60, Pensacola, FL), with an epoch time set at 10 s. Periods where consecutive zeros exceeded 8 min were identified as non-wear time. To be included in the analyses, subjects had to provide at least 5 h of measurements on at least 24 weekdays and at least 7 h of measurements on at least 2 weekends. The accelerometer data were recorded in “count” units of measurement, and PA was categorized into different intensity levels based on these count values (38). The intensity classification standard developed by Zhu et al. for Chinese school children and adolescents was applied to categorize PA into four levels: “sedentary physical activity” (SPA), “light physical activity” (LPA), “moderate physical activity” (MPA), and “vigorous physical activity” (VPA).

## 3 Questionnaire design

### 3.1 Social support (SS) scales

Referring to Daijun et al. (39). The social support dimension of the scale, which assesses its influence on adolescents’ exercise and health behaviors from the perspective of social ecology theory, comprises 4 question items (e.g., “I often participate in PA with my peers”). Participants rated these items on a 7-point Likert scale. The reliability of these items, as measured by Cronbach’s alpha, was found to be 0.711 in this study. Additionally, the validated confirmatory factor analysis (CFA) metrics indicated a very good model fit: χ^2^/df = 0.464, RMSEA = 0.000, RMR = 0.006, GFI = 1, AGFI = 0.997, and CFI = 1.

### 3.2 Autonomous motivation (AM) scales

We used an adapted version of the Spanish (40) of the Exercise Behavior Modification Questionnaire (41) which contains both internal and external motivation.The scale was independently translated into Chinese by the translator, and then the translated questionnaire was discussed until a consensus was reached, resulting in a preliminary Chinese questionnaire with a reliability of 0.817, which is a good reliability, and was therefore chosen for this study. In our study, we focused on intrinsic motivation as a measure of autonomous motivation (e.g., “Because I feel pleasure and satisfaction when I do exercise,” etc.). Participants rated these items on a 7-point Likert scale. The factor analysis (CFA) results indicated a good model fit: χ^2^/df = 3.923, RMSEA = 0.064, RMR = 0.019, GFI = 0.995, AGFI = 0.973, and CFI = 0.993.

### 3.3 Procedures and data analysis

**Figure 1:**
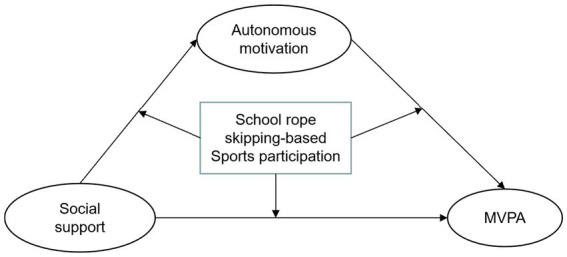
Research architecture.

Initially, we employed SPSS 26.0 (SPSS Inc., Chicago, IL, USA) to input and manage the collected data. Subsequently, we conducted Harman’s one-way method test to assess any potential common method bias. Following this, we performed bivariate correlation analysis to examine relationships among different dimensions and to gauge the strength of correlation between variable factors. Descriptive statistics were used to present the data, expressed as the mean and standard deviation (M ± SD). Subsequently, we employed SEM to assess the relationship between high-intensity PA, potential influencing factors, and pathways in MVPA. Given that there is only one variable for MVPA, it will not be computed when incorporated into structural equation modeling. To address this, we will establish the error variance of the single variable MVPA, which can be determined using the following formula: Error Variance of MVPA = (1 - reliability coefficient) (S2). This approach allows us to account for the single-variable nature of MVPA in the structural equation modeling analysis. If the reliability of scores for X1 is 0.85 with a standard deviation of 5.00, then the error variance of X1 = (1–0.85)(5.00)2 = 0.15(25) = 3.75 (42). In this study, the potential variance single indicator measure MVPA reliability was set at 0.8, and using SPSS descriptive statistics, the MVPA variance (S2) was calculated as 0.328, so the error variance of MVPA in this study = (1–0.8)*0.328 = 0.067. λ0.51 was calculated from the error variance, and then the calculated results were substituted into the model (see Figure 1).

**Figure 2:**
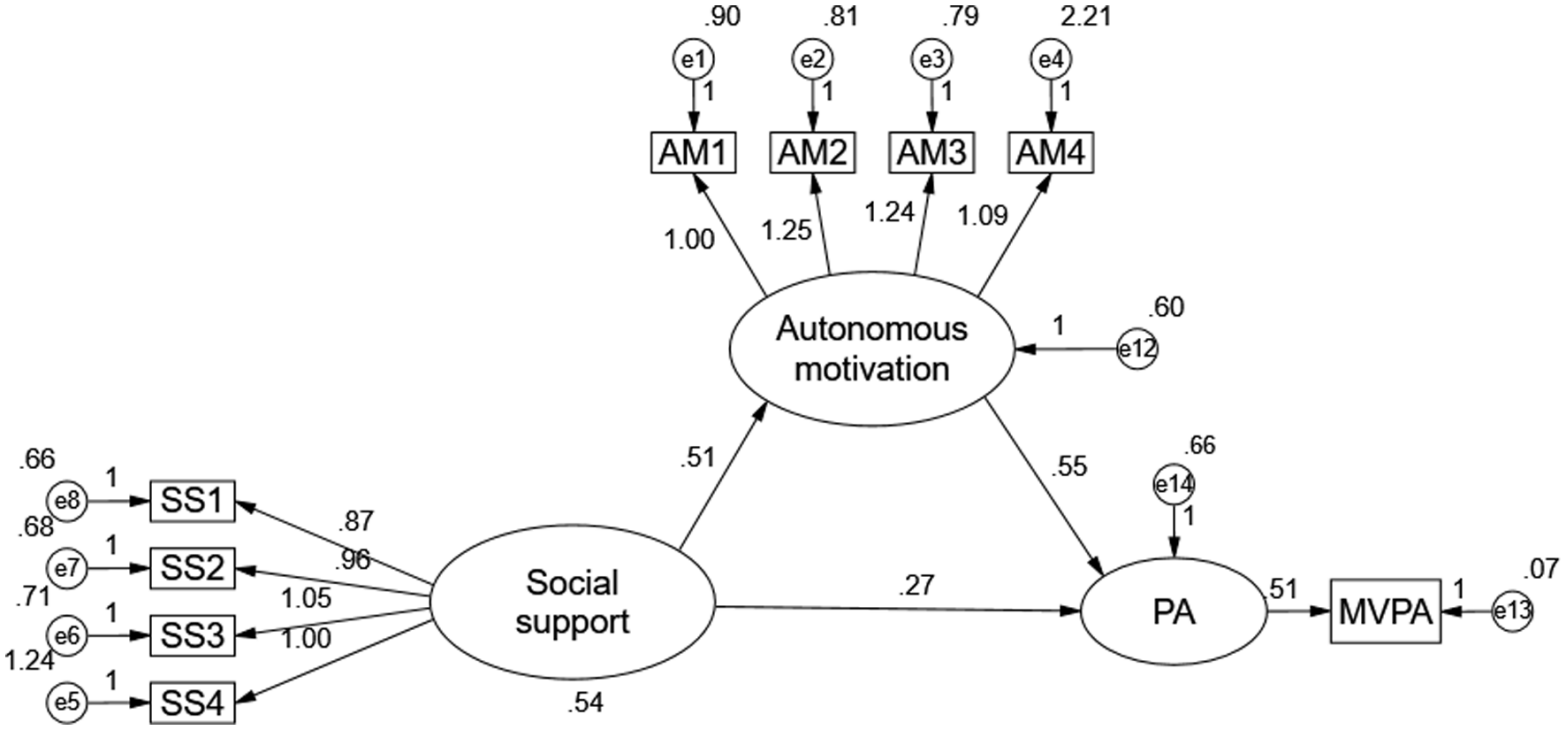
Diagram of a mediated model of PA in school children.

SEM was conducted using AMOS 24.0 (SPSS Inc., Chicago, IL, Unite States) with maximum likelihood estimation. Model fitness was assessed using various indices, which encompassed the ratio of the minimum difference to the degrees of freedom (CMIN/DF), the goodness-of-fit index (GFI), the adjusted goodness-of-fit index (AGFI), the comparative goodness-of-fit index (CFI), and the standardized mean root mean square residual (SRMR) (43, 44). The Bootstrap 5,000 method was employed to perform mediation model tests (refer to Figure 2 and Table 1). These tests encompassed the examination of model direct effects, indirect effects, and total effects. The syntax for direct effects in the structural equations was constructed to evaluate whether social support has a direct impact on school children’s MVPA behavior. Additionally, the syntax for indirect effects was formulated to investigate whether social support indirectly influences school children’s MVPA through autonomous motivation. Confidence intervals obtained from the estimation method were assessed to ascertain whether they contained zero at the 95% confidence level.

**Table 1:** Analysis of the effectiveness of brokering.

Form	Estimate valuation	Product of coefficients		Bootstrapping			
				bias-corrected 95 per cent CI		Percentile 95 per cent CI	
		Z	SE	Lower	Upper	Lower	Upper
Overall effect	0.547	6.924	0.079	0.390	0.707	0.392	0.709
Indirect effect	0.282	5.127	0.055	0.180	0.405	0.179	0.403
Direct effect	0.265	3.118	0.085	0.097	0.435	0.097	0.435

To conduct the moderated effects analysis, we initially computed the standardized values of the variables and the product coefficients of the interaction terms. These standardized values and interaction terms were subsequently incorporated into the model to assess the presence of moderating effects. Specifically, we aimed to determine whether the interaction terms significantly influenced the path coefficient (γ) of the dependent variable and whether the value of p was less than 0.05, indicating statistical significance. The moderated effects plots were generated using the Johnson-Neyman’s method, implemented as an SPSS plug-in within the PROCESS framework. This approach allows for a comprehensive visualization and interpretation of the moderating effects in the analysis.

### 3.4 Standard

This study received approval from the Ethics Committee of Xiangya School of Public Health, Central South University (project number XYGW-2022-44, issued on 20 July 2022). The research was carried out in full compliance with the principles outlined in the Declaration of Helsinki. Before participating in the survey, students were provided with clear information that their responses to the questionnaires would remain anonymous, and participation was entirely voluntary. The content of the questionnaires was treated with utmost confidentiality, and the collected data was exclusively employed for scientific research purposes. All questionnaires were collected in person, and all participants were duly informed about the study’s objectives and characteristics. Furthermore, each participant willingly signed an informed consent form to formally acknowledge their agreement to participate in the study.

## 4 Results and analyses

### 4.1 Basic statistical information

In this study, independent samples t-tests were employed to investigate potential differences between genders concerning social support, autonomous motivation, and MVPA. The findings revealed that gender did not yield statistically significant differences in the dimensions of social support and autonomous motivation at the 0.05 significance level. However, notable disparities emerged among school children in MVPA and SRSP (*p* = 0.003 < 0.01, *p* = 0.013 < 0.05). Specifically, it was observed that boys exhibited a higher level of engagement in PA compared to girls (refer to Table 2).

**Table 2:** Independent samples t-test of different genders and dimensions.

Dimension	Mean equivalence *t*-test				Gender	Number of cases	M	SD
	T	DF	*p*	Mean difference				
Social support					Girl	315	4.628	0.700
	−1.209	717	0.227	−0.074	Boy	406	4.702	0.947
Autonomous motivation	−0.297	719	0.767	−0.025	Girl	315	4.683	1.058
					Boy	406	4.708	1.169
MVPA	−2.935	719	0.003	−0.125	Girl	315	2.759	0.522
					Boy	406	2.884	0.603
SRSB	−2.495	719	0.013	−0.185	Girl	315	3.041	0.056
					Boy	406	3.226	0.048

### 4.2 Common methodological controls and tests

To mitigate potential common methodological biases in the gathered data, a systematic approach was implemented to oversee the measurement process. The Harman one-factor method was specifically employed to examine the presence of common bias. The results of this analysis revealed the existence of two factors with eigenvalues exceeding 1. However, it’s noteworthy that the first factor accounted for only 26.96% of the total variance, which fell below the critical threshold of 40%. This outcome indicates that there was no substantial evidence of common method bias affecting the study’s findings (45).

### 4.3 Correlation analysis of variables

Pearson correlation analyses were diligently executed for each variable, and the resulting correlations are meticulously presented in Table 3. These findings reveal noteworthy patterns of association among the variables. Specifically, it was observed that social support exhibited a significantly positive correlation with autonomous motivation, MVPA, and SRSP (r = 0.331, *p* < 0.01, r = 0.308, *p* < 0.01, r = 0.103, *p* < 0.01). Furthermore, autonomous motivation demonstrated a significant positive correlation with MVPA and (r = 0.459, *p* < 0.01, r = 0.155, *p* < 0.01). Additionally, exhibited a significant positive correlation with MVPA (r = 0.198, *p* < 0.01). These correlations underscore the interconnectedness of these variables within the study’s context.

**Table 3:** Correlation and descriptive statistics of variables in the prediction model.

Dimension (math.)	Social support	Autonomous motivation	MVPA	SRSB	M	SD
Social support	1	0.331^**^	0.308^**^	0.103^**^	4.670	0.848
Autonomous motivation		1	0.459^**^	0.155^**^	4.697	1.121
MVPA			1	0.198^**^	2.893	0.573
SRSB				1	3.146	0.993

**Correlations are significant at the 0.01 level (two-tailed) and the upper triangle is the Pearson correlation of dimensions, *N* = 721.

### 4.4 Analysis of mediating effects of autonomous motivation

As shown in Figure 2, the fit indices of the mediation model are as follows: χ^2^ = 39.807, df = 43, χ^2^/df = 1.592, GFI = 0.988, AGFI = 0.979, CFI = 0.991, NFI = 0.975, TLI = 0.986, RMSEA = 0.029, RMR = 0.024, and the prediction γ of the social support on the autonomous motivation is 0.51, *p* < 0.001. The predicted γ for social support was 0.51, *p* < 0.001, the predicted γ for social support on MVPA was 0.27, *p* < 0.001, and the predicted γ for autonomous motivation on MVPA was 0.55, *p* < 0.001, predicting that autonomous motivation mediates the relationship between social support and children’s MVPA. In order to calculate the mediating effect more accurately, this paper tested the mediating effect with structural equation modelling analysis, firstly using Bootstrap 5,000 estimation technique to estimate the standard error of the mediating effect, and then further calculating the significant level of the mediating effect. The results showed (Table 1) that the total effect of social support on children’s MVPA was 0.547, with a standard error of 0.079 and a Z-value of 6.924, which meets the criterion of greater than 1.96.

At 95% confidence level, the lower limit of the confidence interval obtained by the Bias-corrected estimation method is 0.390, and the upper limit is 0.707, the lower limit of the confidence interval obtained by the Percentile estimation method is 0.392, and the upper limit is 0.709, which does not include zero, so the total effect is established. Similarly, the indirect and direct effects are also valid. The value of indirect effect is 0.282, accounting for 51.6%, and the direct effect is 0.265, accounting for 48.4%. Therefore, autonomous motivation has a mediating effect on social support and MVPA.

### 4.5 A mixed model test of the moderating effects of SRSP

**Figure 3:**
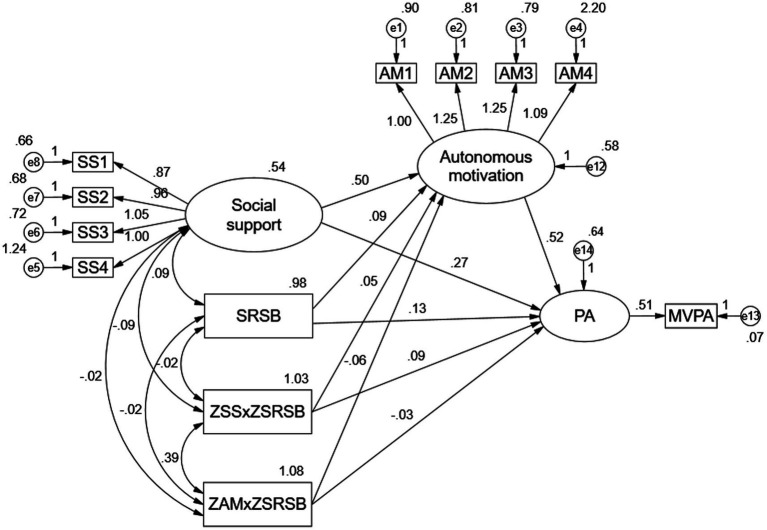
Mixed model of school children’s PA.

SEM was used to examine the mechanisms of social support, SRSP, the interaction between the two aforementioned, and autonomous motivation on children’s MVPA, using great likelihood estimation to test the hypothesised model shown in Figure 3. In this, the interaction terms were standardised and then the multiplication of the interaction terms was calculated and brought into the SEM, if the interaction terms were significant then an interaction existed. In Figure 3, the fit indices of the mixed model are as follows: χ^2^ = 54.098, df = 43, χ^2^/df = 1.258, GFI = 0.988, AGFI = 0.978, CFI = 0.994, NFI = 0.970, TLI = 0.990, RMSEA = 0.019, and RMR = 0.022, which are within the good range of the fit indices. Within the range, so it can be concluded that the data fit the model well. The results showed that the path coefficient γ of the interaction term (social support × SRSP) on children’s MVPA was 0.09, CR = 2.17, *p* = 0.03 < 0.05, indicating that the direct moderating effect of SRSP was significant. The path coefficient γ of the interaction term (social support × SRSP) on autonomous motivation was 0.05, CR = 1.49, *p* = 0.14 > 0.05, indicating that the moderating effect of SRSP on the relationship between social support and children’s MVPA was not established, i.e., the effect of SRSP on the relationship between social support and children’s MVPA could not be achieved through the mediation of autonomous motivation in the first half of the interaction term. Mediated by the first half of the The path coefficient γ of the interaction term (autonomous motivation × SRSP) on children’s MVPA was-0.03, CR = −0.74, *p* = 0.46 < 0.05, indicating that the moderating effect of SRSP on autonomous motivation and children’s MVPA was not established. The path coefficient γ of SRSP on children’s MVPA was 0.13, CR = 3.38, *p* = 0.00 < 0.05, and the path coefficient γ of SRSP on autonomy motivation was 0.09, CR = 2.71, *p* = 0.01 < 0.05. This result verified that the effect of social support on children’s MVPA was moderated by SRSP.

### 4.6 Johnson-Neyman’s diagram of moderating effects

**Figure 4:**
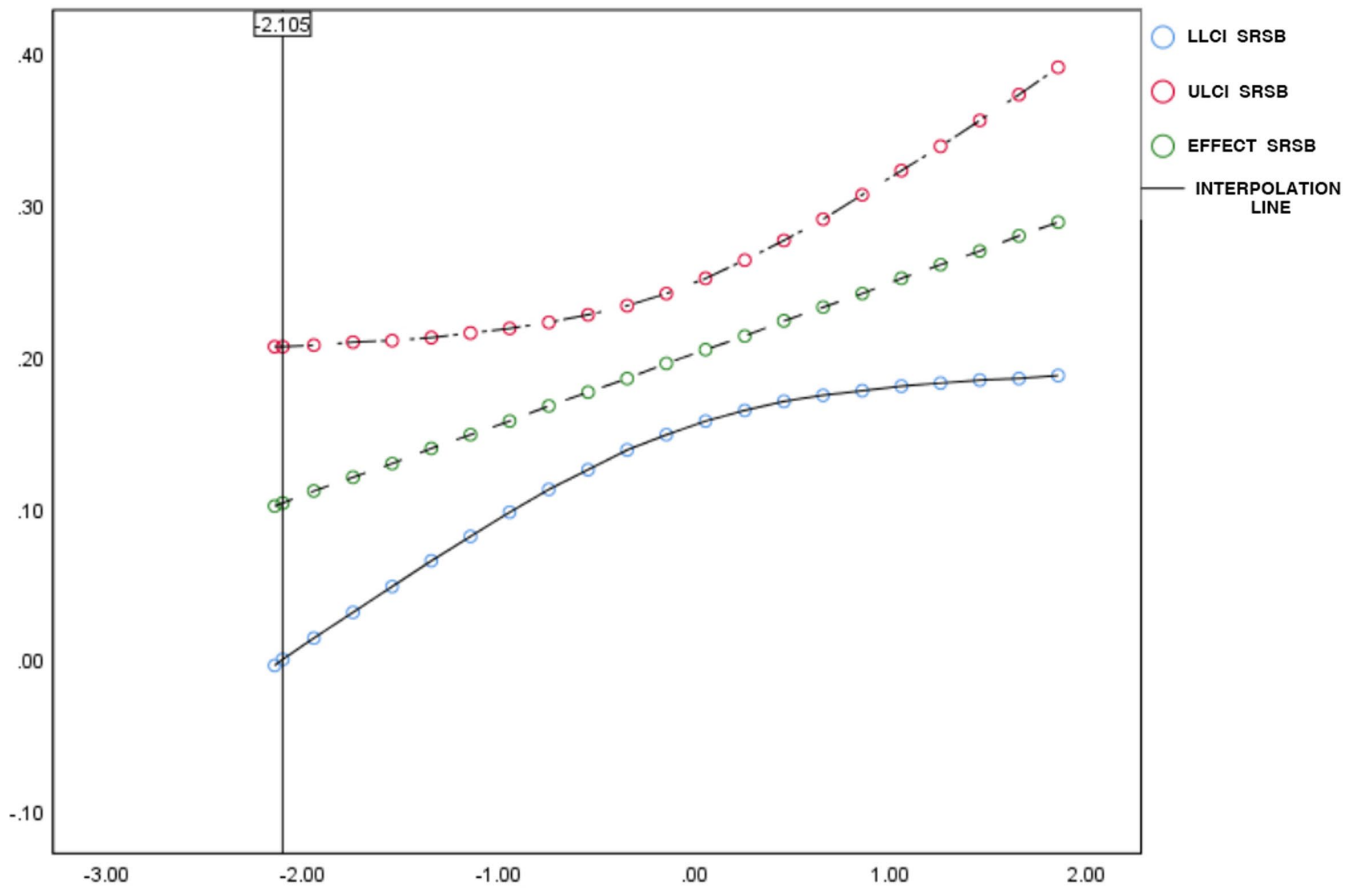
Johnson-Neyman’s test for moderating effects interpolation line. LLCI and ULCI are the lowest and highest values of the confidence interval, respectively, and the interpolation line refers to the dotted line connecting the dots.

To better understand the moderating effect in the relationship between social support and MVPA in school children and identify the conditions under which this moderating effect occurs, the Johnson-Neyman test method was employed. The results, as illustrated in Figure 4, indicate that the moderating effect starts at a value of minus 2.105. Within this interval, there is no significant difference observed on the left side, suggesting that lower levels of SRSP do not significantly moderate the relationship between social support and MVPA in school children.

However, on the right side of the interval, concerning higher levels of SRSP, there is a significant moderating effect observed in the relationship between social support and MVPA in school children. This implies that when school children engage in higher levels of SRSP, the interaction between this participation and social support significantly affects their MVPA.

## 5 Discussion

### 5.1 Social support, autonomous motivation directly explains school children’s MVPA

The findings of this study reveal several significant relationships within the context of school children’s PA. Specifically, the study highlights that both social support and autonomous motivation are positively linked to school children’s MVPA. Moreover, the quality of teacher-student relationships, peer relationships, parental support, and the degree of autonomy school children perceive within their school environment directly impact their emotional experiences. These emotional experiences, in turn, play a pivotal role in shaping school children’s commitment to engaging in MVPA. We found multiple studies examining the relationship between teacher influence and school children’s PA participation. For example, teacher support positively predicted school children’s PA participation, and physical education teacher support in particular positively promoted school children’s participation in PA (46–48). Furthermore, the study suggests that friendships among school children can exert a noteworthy influence on their participation in MVPA. Friends communicate through various means, such as social norms and dialogues related to MVPA. These interactions can involve positive messages, including encouragement and support, which contribute to shaping school children’s attitudes and behaviors towards PA (49–52). The conduct of friends, which can provide guidance to their peers (53–56), includes participating in MVPA with friends, such as organized sports and recreational activities in their company. These activities are considered positive behaviors (55, 57–59). Communication regarding MVPA frequently involves friends providing support and encouragement. This support can be assessed through factors that gauge social support. These factors may include friends reminding individuals to engage in exercise, encouraging them to take part in MVPA, praising their involvement in such activities, or engaging in discussions with them on topics related to MVPA (60). In models exploring communication about MVPA or social support, all twenty-five items exhibited significant positive correlations (57, 60–64). Autonomous motivation has been demonstrated to be linked to increased levels of MVPA in school children and adolescents (65–68).The motivation for MVPA is progressively emerging as a significant factor influencing school children’s mental health. When individuals are autonomously motivated, they tend to achieve better physical and mental outcomes naturally (69, 70). Research has demonstrated that greater levels of autonomous motivation are predictive of increased positive affect in adolescents (71). This study illustrates the beneficial effect of autonomous motivation on sustaining MVPA (72). In this study, the favorable effects of autonomous motivation on the maintenance of MVPA were showcased. Additionally, social support and autonomous motivation played crucial roles in enhancing MVPA levels. To promote MVPA participation in school children and adolescents, interventions aimed at fulfilling psychological needs and fostering positive affective experiences are essential.

### 5.2 Autonomous motivation mediates the relationship between social support and school children’s MVPA

In this study, we assessed and analyzed the mediating role of autonomous motivation in the connection between social support and the MVPA levels of adolescents. Our findings confirm the presence of this mediating relationship. We observed that social support not only has a direct impact on school children’ MVPA but also exerts an indirect influence through autonomous motivation. Notably, there was a significant difference in the indirect effect (Z = 5.127), with a bias-corrected 95% confidence interval [0.180, 0.405] and a percentile-based 95% confidence interval [0.179, 0.403]. This mediation effect amounted to 0.282 or 51.6%. Relevant studies suggest that parental support plays a pivotal role in enhancing school children’s MVPA and influencing their choices and behaviors (73). Encouraging family members to engage in MVPA alongside their school children promotes an increase in MVPA among the school children (74). It has been suggested that social support such as family support (75, 76)and peer support influence motivation to exercise (77), and that social support plays an important role in promoting autonomous motivation for healthy exercise (78) and social support plays an important role in promoting autonomous motivation for healthy exercise. In accordance with the principles of self-determination theory, autonomous motivation encompasses both internal and fully internalized external sources of motivation. It represents an individual’s intrinsic drive to consistently strive towards their exercise-related goals (79). A comprehensive meta-analysis comprising 46 studies that met the specified inclusion criteria revealed a positive correlation between overall levels of autonomous motivation and engagement in MVPA (80), and that motivation is an important correlate and potential determinant of MVPA (81). The current study further corroborated the significant impact of autonomous motivation on MVPA (with a γ coefficient of 0.55, *p* < 0.001). In MVPA scenarios, students exhibit a strong desire to achieve goals related to reducing anxiety, enhancing mood, and fostering both physical and mental well-being through MVPA. They possess an intrinsic and compelling need for MVPA. Conversely, social support assumes a pivotal role as an external catalyst. School children with robust social support tend to experience higher exercise efficacy and a heightened sense of accomplishment. This support allows them to reinforce internal motivational elements, such as the desire for exercise and interest in MVPA, thereby sustaining their motivation and behavior for exercise. In essence, when school children recognize that engaging in MVPA contributes to their competence and self-confidence, they develop an increased need for competence, a heightened interest in exercise, and are more likely to maintain frequent and enduring exercise routines. This, in conjunction with their innate desire for MVPA and external triggers provided by social support, stimulates their autonomous motivation to engage in MVPA.

### 5.3 Moderating effects of SRSP on the relationship between social support and MVPA in school children

The findings of this study revealed that participation in SRSP had a direct moderating influence on the relationship between social support and school children’s engagement in MVPA. This suggests that as the level of SRSP increased, the positive impact of social support on school children’s MVPA became more pronounced. When school children perceived adequate social support, they tended to make optimal choices during MVPA, especially when engaging in SRSP, provided they experienced happiness and excitement throughout the process and were content with the resulting outcomes. Consequently, for these school children who are particularly sensitive to social support due to their urgent psychological need for satisfaction and encouragement during exercise, effective social support exerted a more significant influence on their participation in MVPA.

### 5.4 Significance and limitations of the study

The findings of this prospective study hold significant implications. Firstly, the study underscores the substantial relevance of social support and autonomous motivation in enhancing school children’s MVPA. Factors such as family support, peer support, and intrinsic motivation are all closely tied to school children’s MVPA levels. Therefore, it is important to educate schools and families in order to increase the level of autonomous motivation of schoolchildren and their active participation in MVPA. Secondly, the mediation model analysis offers valuable insights into the pivotal role played by autonomous motivation in mediating the relationship between social support and school children’s MVPA. This insight lays the foundation for potential interventions aimed at boosting autonomous motivation, consequently improving school children’s MVPA behaviors. Therefore, the creation of relevant courses or activities can foster and increase the level of autonomous motivation and promote the adoption of positive strategies to promote the active participation of schoolchildren in MVPA. Lastly, the study validates that SRSP is more likely to impact school children’s engagement in MVPA. Therefore, it is advisable to prioritize interventions targeting groups with higher levels of SRSP to enhance school children’s MVPA levels. The results of the study make it important that social support and autonomous motivation enhance the behavioral impact of school children’s MVPA and, more notably, help school children to raise their level of self-motivation and, consequently, their level of behavior.

In future research, demand-supportive behaviors can be used to improve motivation and levels of PA. In a recent study by Ahmadi et al. a classification system for motivational behaviors was proposed which could serve as an important basis for future research (82). When a person’s psychological needs are met in an activity, the development of intrinsic motivation towards that activity is promoted. This suggests that in order to promote PA in children, interventions can be used to support their basic psychological needs in the context of PA (83).

This study has several limitations. Firstly, its cross-sectional design restricts the ability to establish causality, as causal relationships cannot be inferred. Future research should employ a longitudinal research design to further validate the causal hypotheses proposed in this study. Secondly, the study exclusively focused on the impacts of social support and autonomous motivation on school children’s MVPA. It did not consider other potential mediating factors such as sensation seeking and psychological resilience, which might also influence school children’s MVPA. Therefore, future research should explore the interplay between these variables to gain a more comprehensive understanding of their relationships. Lastly, this study employed whole cluster random sampling and collected data from only one district. Consequently, the results may not be readily generalizable to a broader population. To enhance the generalizability of the findings, future studies should employ more diverse and representative sampling methods to encompass a broader spectrum of school children, including those from different regions in China.

## 6 Conclusion

Social support and autonomous motivation were both direct predictors of school children’s MVPA. Additionally, social support exerted an indirect influence on school children’s MVPA through the intermediary role of autonomous motivation. And autonomous motivation played a crucial “bridging” role in mediating the relationship between social support and school children’s MVPA. Besides, the interaction between social support and participation in SRSP had a moderating effect on the levels of MVPA in school children. Improvements in social support and autonomous motivation are conducive to an environment that enhances healthy behaviors and overall MVPA for school children, and future efforts should be directed at continuing to improve social support and autonomous motivation for school children, and enhancing psychological access for school children. Overall, this study provides ideas for the future development of physical education policies in schools.

## Data availability statement

The raw data supporting the conclusions of this article will be made available by the authors, without undue reservation.

## Ethics statement

The studies involving humans were approved by Xiangya School of Public Health, Central South University (No XYGW-2022-44; 20 July 2022). The studies were conducted in accordance with the local legislation and institutional requirements. Written informed consent for participation in this study was provided by the participants’ legal guardians/next of kin. Written informed consent was obtained from the individual(s), and minor(s)’ legal guardian/next of kin, for the publication of any potentially identifiable images or data included in this article.

## Author contributions

YQ: Conceptualization, Funding acquisition, Methodology, Project administration, Supervision, Validation, Writing – original draft. YY: Data curation, Formal analysis, Software, Writing – original draft. XW: Investigation, Visualization, Writing – review & editing. YZ: Resources, Supervision, Visualization. BL: Resources, Funding acquisition, Writing – review & editing.

## Supplementary material

The Supplementary material for this article can be found online at:

https://www.frontiersin.org/articles/110.3389/fpubh.2024.1295924/full#supplementary-material
