# Supplementary material for: Autonomous motivation, social support, and physical activity in school children: moderating effects of school-based rope skipping sports participation
Source: Front Public Health. 2024 Jan 24;12:1295924. doi: 10.3389/fpubh.2024.1295924 (PMC10847259; doi:10.3389/fpubh.2024.1295924)
Supplement: Supplementary file 1 [file Table_1.DOCX]

#### Informed Consent Form (parent version)

Dear Parents.

Hello! In order to promote healthy child development, we would like to invite you to participate in a Hunan Provincial Education Science Planning Project. Project title: A cluster randomised controlled trial of a school gardening, cooking and sports participation intervention to improve fruit and vegetable intake and moderate-to-vigorous physical activity in Chinese school-age children.

Before you decide whether to take part in this study, please read the following as carefully as possible. It will help you understand the study and why it is being conducted, the procedures, the benefits, risks and discomforts you may experience as a result of participating in the study.

**I. Purpose of the study**

To investigate the current status of vegetable and fruit intake among school-age children in public primary schools in Changsha and to analyse the effects of moderate-to-vigorous physical activity, sedentary hours and sleep duration.

**II. What do you need to do if you participate in the study?**

1. you will be required to complete an electronic questionnaire by scanning a QR code via your smartphone.

2. your child will participate in physical measurements (e.g. height and weight).

3. Depending on your wishes, your child will have the opportunity to participate in wearing the Huawei Sports Bracelet 6 to measure compliance with various lifestyle indicators.

**III. Benefits of participating in the trial**

1. We will provide your child's primary school with nutritional recipes based on the child's nutritional developmental needs.

2. We will take your child's physical measurements and analyse your child's growth and development. For children who are underweight, overweight or obese, we will provide professional dietary advice and nutritional guidance.

3. If your child participates in the Huawei Sports Bracelet lifestyle test, you will also receive a lifestyle analysis report.

**IV. Possible risks and discomfort**

During the study, you will be required to complete the questionnaire entries and assist the child in recording the lifestyle situation at home for four days, which may take up a small amount of your time in caring for the child.

**V. Confidentiality of personal information**

All information for this study is collected anonymously. All data collected will only be used for scientific research and will not be disclosed in any way to any entity or individual. Every effort will be made to protect the security of your personal data to the extent permitted by law.

If you have any questions about this study, you can ask any member of staff at the survey site and we will answer them as soon as possible. You can voluntarily choose whether or not to participate in this study, or to withdraw from the study at any time during the course of the study, without any loss to you.

I have read the above, fully understand the possible risks and benefits of participating in this study, and agree to participate in this study.

This informed consent has been reviewed by the Ethics Committee of Xiangya School of Public Health, Central South University (No.: __________, date of ethical review passed _________, effective date: _________).

□ Consent to participate in this study □ Refusal to participate in this study

Date： _______ ______ _____

#### Informed Consent Form (Student Version)

Dear Student.

Hello! In order to promote your healthy development, we would like to invite you to participate in a Hunan Provincial Education Science Planning Project. Project name: A cluster randomised controlled trial of a school gardening, cooking and exercise participation intervention on improving fruit and vegetable intake and moderate-to-vigorous physical activity in Chinese school-age children.

Before you decide whether to participate in this study, please read the following as carefully as possible. It will help you to understand the study and why it is being conducted, the procedures, the benefits, risks and discomforts you may experience as a result of participating in the study.

**I. Purpose of the study**

To investigate the current status of vegetable and fruit intake among school-age children in public primary schools in Changsha and to analyse the effects of factors such as moderate-to-vigorous physical activity, sedentary hours and sleep duration.

**II. What do you need to do if you participate in the study?**

1. You will be required to complete a paper version of the questionnaire.

2、 You will participate in physical measurements (e.g. height and weight).

3. Depending on your wishes, you will have the opportunity to participate in wearing the Huawei Sports Bracelet 6 to measure the attainment of various lifestyle indicators.

**III. Benefits of participating in the trial**

1. We will provide you with nutritional recipes for your primary school based on the nutritional developmental needs of children.

2. We will take your physical measurements and analyse your growth and development status. For children who are underweight, overweight or obese, we will provide professional dietary advice and nutritional guidance.

3. If you participate in the Huawei Sports Bracelet lifestyle test, you will also receive a lifestyle compliance analysis report.

**IV. Possible risks and discomforts**

During the study period, you will be required to complete the questionnaire entries and record your lifestyle at home for four days, which may take up a small amount of your time and may cause you inconvenience.

**V. Confidentiality of personal information**

All information for this study is collected anonymously. All data collected will only be used for scientific research and will not be disclosed in any way to any entity or individual. Every effort will be made to protect the security of your personal data to the extent permitted by law.

If you have any questions about this study, you can ask any member of staff at the survey site and we will answer them as soon as possible. You can voluntarily choose whether or not to participate in this study, or to withdraw from the study at any time during the course of the study, without any loss to you.

I have read the above, fully understand the possible risks and benefits of participating in this study, and agree to participate in this study.

This informed consent has been reviewed by the Ethics Committee of Xiangya School of Public Health, Central South University (No.: __________, date of ethical review passed _________, effective date: _________).

□ Consent to participate in this study □ Refusal to participate in this study

Date： _______ ______ _____
